# Diagnostic Value of Angiography-Derived IMR for Coronary Microcirculation and Its Prognostic Implication After PCI

**DOI:** 10.3389/fcvm.2021.735743

**Published:** 2021-10-15

**Authors:** Neng Dai, Wenliang Che, Lu Liu, Wen Zhang, Guoqing Yin, Bin Xu, Yawei Xu, Shaofeng Duan, Haojun Yu, Chenguang Li, Kang Yao, Dong Huang, Junbo Ge

**Affiliations:** ^1^Department of Cardiology, Shanghai Institute of Cardiovascular Diseases, Zhongshan Hospital, Fudan University, Shanghai, China; ^2^National Clinical Research Center for Interventional Medicine, Shanghai, China; ^3^Department of Cardiology, Shanghai Institute of Cardiovascular Diseases, Shanghai Tenth People's Hospital, Tongji University, Shanghai, China; ^4^GE Healthcare China, Shanghai, China; ^5^Department of Nuclear Medicine, Zhongshan Hospital, Fudan University, Shanghai, China

**Keywords:** coronary microcirculation, index of microcirculatory resistance, prognosis, INOCA, functional angiography

## Abstract

**Background:** Angiography-derived index of microcirculatory resistance (angio-IMR) is an emerging pressure-wire-free index to assess coronary microvascular function, but its diagnostic and prognostic value remains to be elucidated.

**Methods and Results:** The study population consisted of three independent cohorts. The internal diagnostic cohort enrolled 53 patients with available hyperemic microcirculatory resistance (HMR) calculated from myocardial blood flow and pressure. The external diagnostic cohort included 35 ischemia and no obstructive coronary artery disease (INOCA) patients and 45 controls. The prognostic cohort included 138 coronary artery disease (CAD) patients who received PCI. Angio-IMR was calculated after the estimation of angiography-derived fractional flow reserve (angio-FFR) using the equation of angio-IMR = estimated hyperemic Pa × angio-FFR × [vessel length/(K × V_diastole_)]. The primary outcome was a composite of cardiac death or readmission due to heart failure at 28 months after index procedure. Angio-IMR demonstrated a moderate correlation with HMR (R = 0.74, *p* < 0.001) and its diagnostic accuracy, sensitivity, specificity, and area under the curve to diagnose INOCA were 79.8, 83.1, 78.0, and 0.84, respectively, with a best cut-off of 25.1. Among prognostic cohort, patients with angio-IMR ≥25.1 showed a significantly higher risk of cardiac death or readmission due to heart failure than those with an angio-IMR <25.1 (18.6 vs. 5.4%, adjusted HR 9.66, 95% CI 2.04–45.65, *p* = 0.004). Angio-IMR ≥25.1 was an independent predictor for cardiac death or readmission due to heart failure (HR 11.15, 95% CI 1.76–70.42, *p* = 0.010).

**Conclusions:** Angio-IMR showed a moderate correlation with HMR and high accuracy to predict microcirculatory dysfunction. Angio-IMR measured after PCI predicts the risk of cardiac death or readmission due to heart failure in patients with CAD.

**Clinical Trial Registration:** Diagnostic and Prognostic Value of Angiography-derived IMR (CHART-MiCro), NCT04825028.

## Introduction

Percutaneous coronary intervention (PCI) is one of the important treatments for coronary artery disease (CAD) and aims to increase myocardial blood flow (MBF). However, it has been reported that 20–60% of patients still experience recurrent angina after PCI (1), which was partly attributed to microcirculatory dysfunction. Several studies have shown that microvascular dysfunction is an important factor that is related with adverse outcomes in CAD patients. The first step to the successful management of such condition is early identification and diagnosis. Although non-invasive imaging modalities including positron emission tomography and cardiac magnetic resonance were optimal for microcirculatory dysfunction assessment, they are not available at the cardiac catheterization laboratory during PCI. Invasive assessments, such as the index of microcirculatory resistance (IMR) and hyperemic microvascular resistance (HMR), have been validated as good indices (2, 3) for the quantitative measurement of coronary microcirculatory dysfunction. However, additional procedural time/complexity, increased procedural cost, and the need for maximal hyperemia may prohibit their usage in clinical practice.

With the technical development, angiographic derivation of fractional flow reserve (FFR) or IMR (angio-IMR), which does not require pressure wire, hyperemic agents, or theromdilution method, is proposed recently as a potential alternative for pressure wire–derived FFR or IMR (4, 5).

In this regard, our study aimed to evaluate the diagnostic performance of angio-IMR for microcirculatory dysfunction and its prognostic implication after PCI in stable CAD.

## Methods

### Study Population

**Figure 1:**
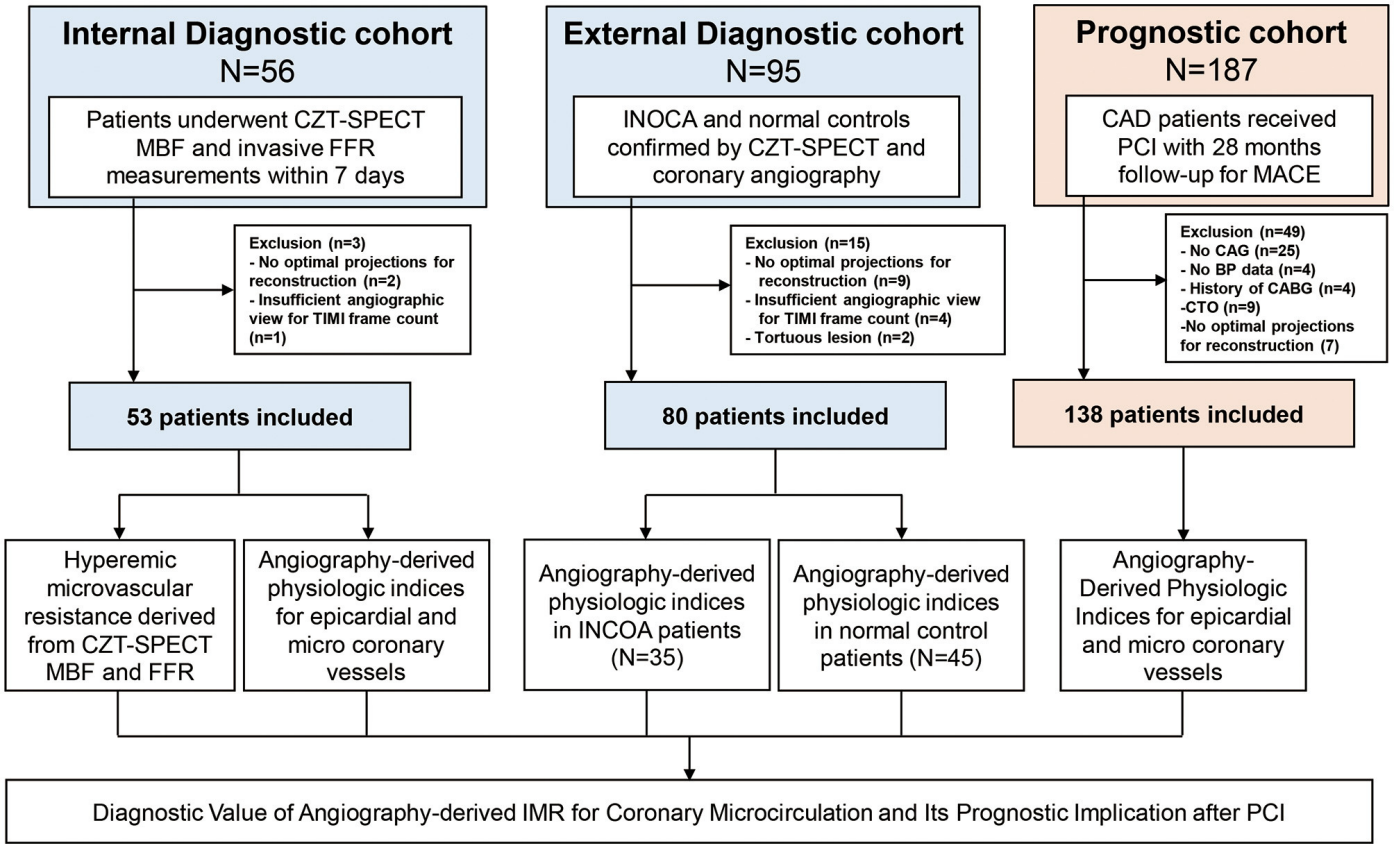
Study flow. The study population was composed of internal and external diagnostic cohorts and prognostic cohort. Internal diagnostic cohort was used to evaluate the correlation between angio-IMR and HMR; external diagnostic cohort was used to evaluate the diagnostic performance of angio-IMR to diagnose INOCA. Prognostic cohort was used to evaluate the prognostic implication of angio-IMR in CAD patients after PCI. Angio-IMR, angiography-derived index of microcirculatory resistance; CAD, coronary artery disease; HMR, hyperemic microcirculatory resistance; INOCA, ischemia and no obstructive coronary artery disease; PCI, percutaneous coronary intervention.

The study population was composed of three independent cohorts (Figure 1). Patients in the internal diagnostic cohort were selected from Zhongshan Hospital, which consisted of 53 consecutive patients with available cadmium–zinc–telluride single-photon emission computed tomography (CZT-SPECT) within 7 days of FFR measurement in the left anterior descending coronary artery. External diagnostic cohort–enrolled patients received CZT-SPECT and invasive angiography examinations for conventional clinical practice from Shanghai Tenth People's Hospital, whose results were previously published (6). Among this cohort, 35 patients with ischemia and no obstructive coronary artery disease (INOCA) were included; 45 patients with no obstructive CAD and normal CZT-SPECT perfusion imaging were regarded as normal controls, while vessels with normal corresponding perfusion territory in INOCA patients were regarded as internal controls. The prognostic cohort included 138 consecutive CAD patients who received PCI with available angiograms and who were suitable for angiography-derived FFR (angio-FFR) and angio-IMR measurements. The primary clinical outcome was cardiac death or readmission due to heart failure at a median of 28 months after the index procedure.

The institutional review board or ethics committee at each participating center approved the current study protocol, which was in accordance with the Declaration of Helsinki. In addition, written informed consent was obtained from all participants.

### CZT- SPECT Perfusion Imaging and Analysis

CZT-SPECT perfusion imaging was performed using a dedicated cardiac scanner (Spectrum Dynamics, Caesarea, Israel). The single-day rest/stress imaging protocol was applied as described before (7).

In the internal diagnostic cohort, full scanning was conducted after establishing the scanning region of interest (ROI). For stress imaging, adenosine triphosphate (ATP) disodium was intravenously administrated at a rate of 140 μg·kg^−1^·min^−1^ for 5 min to induce pharmacological stress, followed by dynamic image acquisition. Rest and stress dynamic images were reconstructed into 32 time frames (21 × 3, 1 × 9, 1 × 15, 1 × 21, 1 × 27, and 7 × 30 s) for dynamic perfusion analysis. Using the previously established Renkin–Crone equation for ^99m^Tc-sestamibi (MIBI), the MBF can be extrapolated from time–activity curves by inputting uptake rate K1 (8). The global myocardial ROI was divided into three regional ROIs corresponding to coronary territories of the left anterior descending coronary artery, right coronary artery, and left circumflex coronary artery, respectively, and the regional MBF was extrapolated for each coronary territory.

In the external diagnostic cohort, visual assessment for the stress and rest perfusion images was performed using the 17-segment model of the left ventricle and a five-point scale (0 = normal, 1 = equivocal, 2 = moderate, 3 = severe reduction of radioisotope uptake, 4 = absence of detectable radiotracer activity in a segment) (9). The summed stress score (SSS) and summed rest score (SRS) are the sum of all defects on the stress and rest image, respectively. The summed difference score (SDS) is defined as the difference between SRS and SSS. Myocardial ischemia in individual coronary territories was defined when the SSS was ≥4 and SDS was ≥2 (10).

Two experienced nuclear physicians who were blinded to the clinical data and angiography or wire-derived physiologic indices analyzed the images using Corridor 4DM software (INVIA, Ann Arbor, MI, USA) and QPS software (Cedars-Sinai Medical Center, Los Angeles, CA, USA).

### Coronary Angiography

Coronary angiography was performed with standard techniques. All angiograms from the internal and the external diagnostic cohorts were recorded at 15 frames per second and analyzed at core laboratory (Zhongshan Hospital, Fudan University, Shanghai Institute of Cardiovascular Diseases, Shanghai, China) in a blinded fashion. Anatomical parameters including minimal lumen diameter, reference vessel size, lesion length, and percent diameter stenosis were analyzed, on the basis of quantitative coronary angiography (QCA) using the QAngio XA software package (Medis Medical Imaging Systems, Leiden, Netherlands).

### Coronary Physiological Measurements and Calculations

Five thousand international units of intravenous heparin and intracoronary nitroglycerin were administrated before invasive FFR measurements. A 0.014-in. coronary pressure wire (Pressure Wire X; Abbott Vascular, Santa Clara, CA, United States) equipped with pressure sensor was advanced to the distal segment of a target vessel after equalization. Maximal hyperemia was induced through intravenous ATP (140 μg·kg^−1^·min^−1^) administration. After measurements, the pressure wire was pulled back to the guide catheter to identify possible pressure drift. FFR was calculated as the mean distal coronary pressure (Pd) divided by the mean aortic pressure (Pa) during maximal hyperemia. FFR ≤0.8 was considered positive for ischemia.

**Figure 2:**
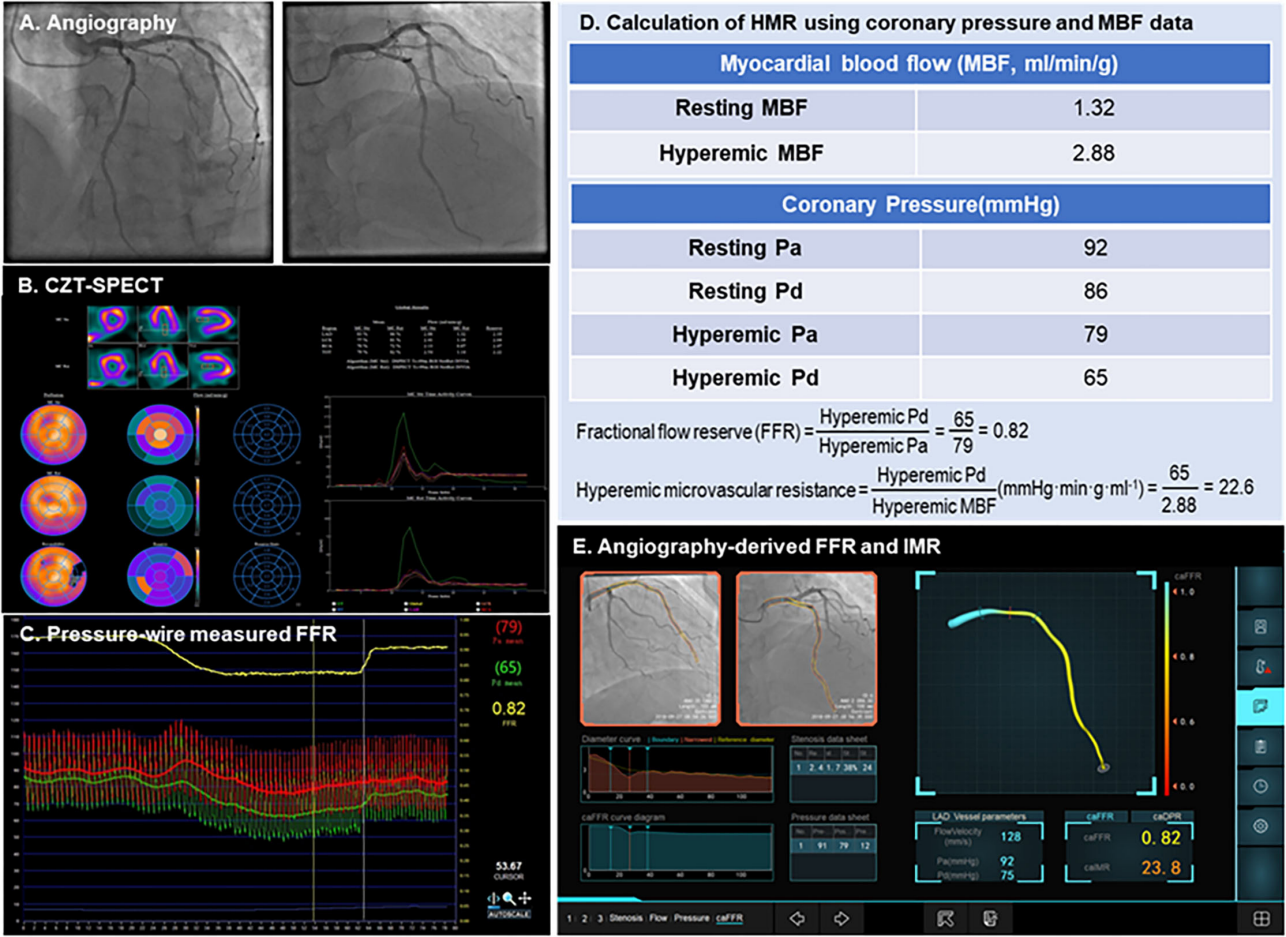
Case examples of angiography-derived physiologic indices. A representative case of CZT-SPECT MBF, pressure wire FFR, and angio-IMR measurements, as well as how HMR are calculated from MBF and coronary pressure, are shown. **(A)** Coronary angiography; **(B)** CZT-SPECT MBF; **(C)** Pressure wire– measured FFR; **(D)** Calculation of HMR using coronary pressure and MBF data; **(E)** Angiography-derived FFR and IMR. CZT-SPECT, cadmium–zinc–telluride single-photon emission computed tomography; FFR, fractional flow reserve; HMR, hyperemic microcirculatory resistance; IMR, index of microcirculatory resistance; MBF, myocardial blood flow.

HMR was calculated as the ratio of hyperemic Pd to hyperemic MBF (Figure 2) (11). The rate–pressure products did not differ significantly at the time point of CZT-SPECT and invasive FFR assessments (8,926.2 vs. 8,784.7 mmHg·bpm; *p* = 0.375), demonstrating no myocardial oxygen demand change over the gap between two examinations.

### Angio-Derived FFR and IMR Measurements

The angio-IMR measurement was conducted as described before using commercialized software (FlashAngio, Rainmed Ltd., Suzhou, China) (12). In brief, a three-dimensional reconstruction of coronary arteries was firstly conducted for the target vessels, followed by the estimation of angio-FFR by computational pressure–flow dynamics with a validated method (5); the estimated hyperemic Pa (Pa_hyp_) was assumed by mean arterial pressure (MAP) during the index procedure–MAP × 0.2 when MAP ≥95 mmHg or MAP-MAP × 0.15 when MAP <95 mmHg (5).

Thus, angio-IMR was calculated as

$$\begin{array}{l} Angio-IMR=Pd_{hyp}\frac{L}{kV_{diastole}} \end{array}$$

where L represents the length from the inlet to the distal position, Pd_hyp_ is the mean pressure (unit: mmHg) at the distal position at the maximal hyperemia, which is computed by the FlashAngio software as the product of Pa_hyp_ and angio-FFR, V_diastole_ is the mean flow velocity (unit: mm/s) at the distal position at diastole, and K is a constant (K = 2.1) obtained from a previous literature (13).

The analysis of angio-IMR was performed by an independent core laboratory (Zhongshan Hospital, Fudan University, Shanghai Institute of Cardiovascular Diseases, Shanghai, China) in a blinded fashion for clinical data, CZT-SPECT, and wire-derived physiologic indices.

### Data Collection, Follow-Up, and Outcomes

Demographic data and cardiovascular risk factors were retrospectively collected. Outpatient visits or telephone contacts were performed every 2 months. The median follow-up duration of the prognostic cohort was 28 months (Q1–Q3 10.3–36.0).

The primary outcome of the prognostic cohort was major adverse cardiac events (MACE) including cardiac death and readmission due to heart failure. All clinical outcomes were defined according to the Academic Research Consortium report (14). The secondary outcomes were a composite of cardiac death, readmission due to heart failure and angina, a composite of cardiac death, readmission due to heart failure, spontaneous myocardial infarction (MI), target vessel revascularization and readmission due to angina, and the individual components of these adverse outcomes. Cardiovascular death was defined as death due to myocardial infarction, significant cardiac arrhythmia, refractory heart failure, or cardiogenic shock. Readmission due to heart failure was defined as hospitalization due to new or worsening signs and symptoms of heart failure in conjunction with non-invasive imaging finding or increased N-terminal pro B-type natriuretic peptide concentrations and a discharge diagnosis of congestive heart failure. Readmission due to angina was defined according to the Braunwald Unstable Angina Classification and the Canadian Cardiovascular Society Angina Classification. Spontaneous MI was defined as an elevation of creatine kinase–myocardial band or a troponin level greater than the upper limit of normal with concomitant ischemic symptoms or electrocardiography findings indicative of ischemia (15). Ischemia-driven target vessel revascularization was defined as a revascularization procedure with at least one of the following: (1) recurrence of angina; (2) positive non-invasive test; and (3) positive invasive physiological test. All events were adjudicated by an expert of interventional cardiology in a blinded fashion.

### Statistical Analysis

Categorical variables are presented as numbers and relative frequencies (percentages); continuous variables are presented either as mean ± SD or median with interquartile range (IQR) according to their distributions, which were checked by using the Kolmogorov–Smirnov and Levene tests. In the internal diagnostic cohort, correlation coefficients were calculated to assess the relationship between angio-FFR and FFR or between angio-IMR and HMR (Pearson or Spearman according to the normality).

In the external diagnostic cohort, the diagnostic performances (including sensitivity, specificity, positive predictive value, negative predictive value, diagnostic accuracy, positive likelihood ratio, and negative likelihood ratio) of angio-IMR to predict INOCA were assessed. The area under curve (AUC) in the receiver-operator characteristic (ROC) curve was calculated for angio-IMR, and the optimal cutoff value of angio-IMR to predict INOCA was calculated to maximize the product of sensitivity and specificity using ROC curves. Intra-individual variability was assessed by two repeated measurements of angio-IMR and angio-FFR with time interval.

In the prognostic cohort, the cumulative incidence of clinical events was presented as Kaplan–Meier estimate and compared using a log-rank test. Multivariable Cox proportional hazard regression was used to calculate the adjusted hazard ratio (HR) and 95% confidence interval (CI) to compare the risk of clinical events according to angio-IMR. The adjusted covariables were age, sex, left ventricular ejection fraction (LVEF), and post-PCI angio-FFR values. The assumption of proportionality was assessed graphically by log-minus-log plot, and the Cox proportional hazard models for all clinical outcomes satisfied the proportional hazards assumption. The best cut-off value of angio-IMR to predict the risk of cardiac death or readmission due to heart failure was evaluated by the maximally selected log-rank statistics method. Independent predictors for cardiac death or readmission due to heart failure were evaluated by the multivariable Cox proportional hazard regression model, and the discriminant function of predictive model was evaluated with Harrell's c-statistics with 95% CI. The additive prognostic implications of angio-IMR into the model with clinical risk factors were evaluated by assessing improvement in the discriminant and reclassification ability of the models with angio-IMR compared with a reference model with clinical risk factors (age, sex, diabetes, hyperlipidemia, hypertension, LVEF, and post-PCI angio-FFR values), using the category-free net reclassification index (NRI) and integrated discrimination improvement (IDI).

All probability values were two-sided, and *p*-values <0.05 were considered statistically significant. All statistical analyses were performed using R version 4.0.2 (R Foundation for Statistical Computing, Vienna, Austria) and SPSS 25.0 for Windows (SPSS-PC, Chicago, IL, USA).

## Results

### Characteristics of Patients and Lesions in Internal and External Diagnostic Cohort

Figure 1 shows the study flow. Patient and lesion characteristics of internal and external diagnostic cohorts were shown in Supplementary Table 1. In the internal diagnostic cohort, the mean age was 63.5 ± 9.4 and 90.6% were male. The mean angio-FFR in this cohort was 0.84 ± 0.06, and the mean angio-IMR was 24.7 ± 3.2. The pressure wire–measured FFR was 0.81 ± 0.06, and the CZT-SPECT-measured hyperemic MBF was 1.94 ± 0.43, leading to a mean calculated HMR of 40.29 ± 10.94.

In the external diagnostic cohort (Supplementary Table 2), the median SSS and SDS were 4 ± 2 and 3 ± 2, respectively, in INOCA patients. The mean angio-FFR and angio-IMR were 0.94 ± 0.03 and 35.83 ± 13.35 in INOCA patients and 0.93 ± 0.03 and 23.7 ± 9.0 in normal controls.

### Diagnostic Performance of Angio-IMR

Figure 2 shows a case example whose CZT-SPECT MBF was measured within 7 days of pressure wire FFR measurement and summarizes how HMR was calculated from coronary pressures and absolute MBF and how angio-IMR was calculated from an angiogram.

**Figure 3:**
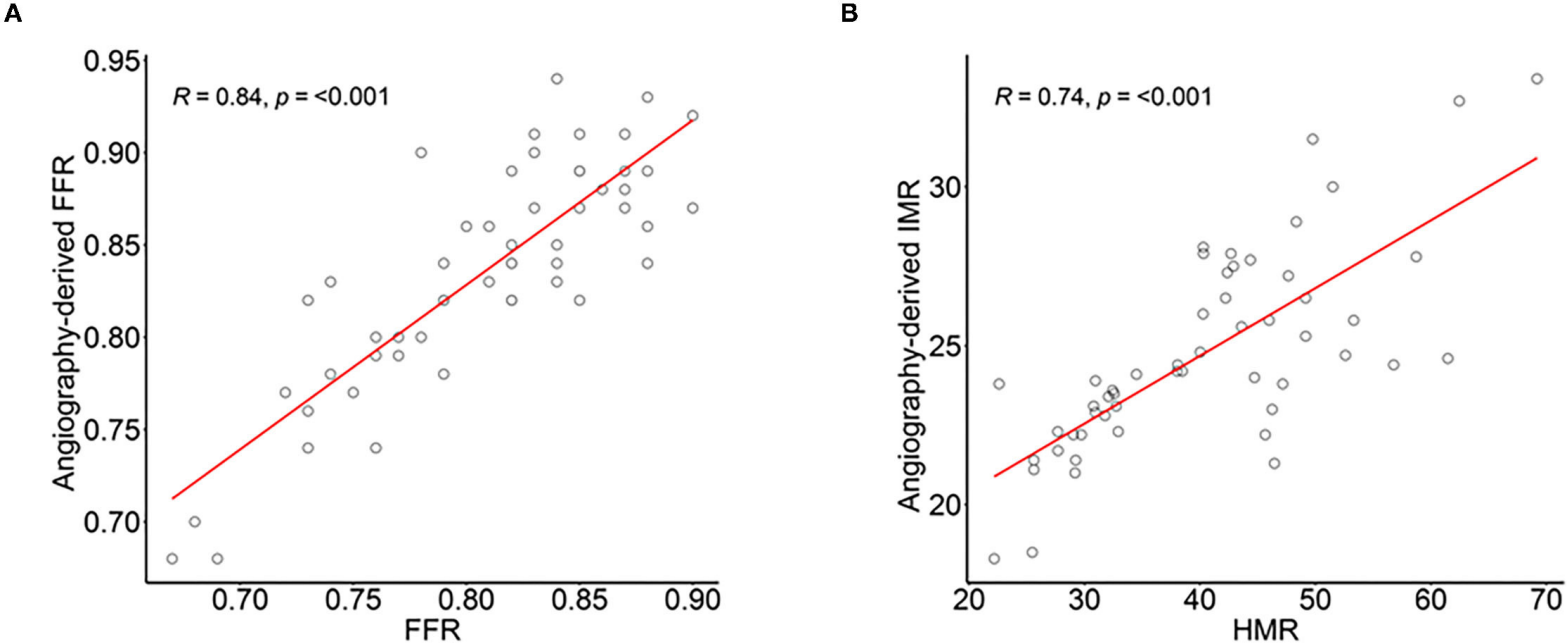
Correlation of angiography-derived FFR with invasive FFR and angiography-derived IMR and HMR in diagnostic cohort. The correlation and agreement between **(A)** angio-FFR and pressure wire–derived FFR, and **(B)** angio-IMR and coronary pressure and myocardial blood flow calculated HMR. Abbreviations are listed in Figures 1, 2.

As shown in Figure 3, there were significant correlations of angio-FFR with FFR (R = 0.84, *p* < 0.001) and angio-IMR with HMR (R = 0.74, *p* < 0.001) and two repeated measures of angio-FFR (R = 0.99, *p* < 0.001) and angio-IMR (R = 0.92, *p* < 0.001) were significantly correlated and nearly the same without significant differences (the differences between two angio-FFR and angio-IMR measurements were 0.002 ± 0.014 and 0.258 ± 2.141, respectively) (Supplementary Figure 1).

**Figure 4:**
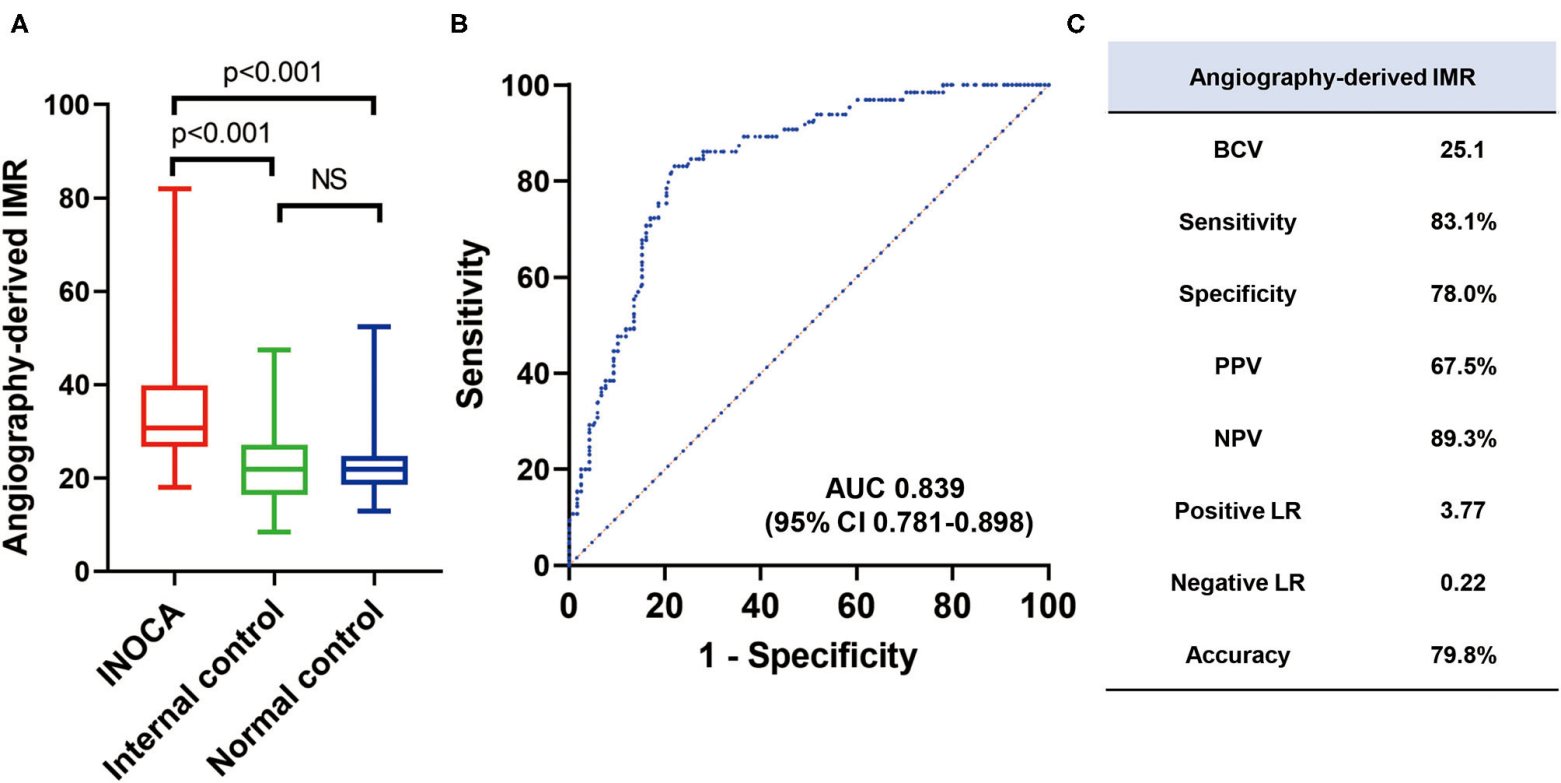
Diagnostic accuracy of angiography-derived IMR to diagnose INOCA in external validation cohort. **(A)** Mean angio-IMR values in vessels with abnormal (INOCA) and normal (internal controls) corresponding CZT-SPECT perfusion territory among INOCA patients and in vessels among patients with normal CZT-SPECT perfusion imaging and angiography (normal controls); **(B)** ROC of angio-IMR to diagnose INOCA and **(C)** diagnostic performances of angio-IMR to diagnose INOCA are shown. AUC, area under curve; BCV, best cut-off value; LR, likelihood ratio; NPV, negative predictive value; PPV, positive predictive value; others are with Figure 1.

The angio-IMR in vessels with abnormal corresponding perfusion territory in INOCA patients was significantly higher than that in the normal corresponding perfusion territory (35.8 ± 13.3 vs. 22.0 ± 7.8, *p* < 0.001) in INOCA patients, as well as that in normal controls (35.8 ± 13.3 vs. 23.2 ± 7.3, *p* < 0.001). ROC analysis demonstrated that angio-IMR had a cut-off value of 25.1 to predict patients with microcirculatory dysfunction represented by normal coronary angiogram but abnormal CZT-SPECT perfusion imaging and showed an AUC of 0.839 (95% CI 0.781–0.898). Sensitivity, specificity, positive predictive value, negative predictive value, and the diagnostic accuracy of angio-IMR were 83.1, 78.0, 67.5, 89.3, and 79.8%, respectively (Figure 4).

### Characteristics of Patients and Lesions in Prognostic Cohort

Table 1 shows patient and lesion characteristics of the prognostic cohort. Mean age was 65.0 ± 8.7, and 96 of the 138 included patients were male. Among these patients, the left anterior descending artery was the most frequent vessel that received PCI. Forty-five (32.6%) patients showed significant microcirculatory dysfunction by angio-IMR ≥25.1. There were no significant differences between angio-IMR <25.1 vs. ≥25.1 regarding patient, lesion, procedural characteristics, and discharge medications.

**Table 1:** Patient and lesion characteristics of prognostic cohort.

	**Total**	**Angiography-derived IMR <25.1**	**Angiography-derived IMR ≥25.1**	***p*-values**
**Patients' characteristics**	138	93 (67.4%)	45 (32.6%)	
***Demographics***				
Age (years)	65.0 ± 8.7	64.9 ± 9.0	65.2 ± 8.1	0.85
Male	96 (69.6%)	69 (74.2%)	27 (60.0%)	0.09
Body mass index (kg/m^2^)	24.6 ± 3.0	24.5 ± 2.8	25.0 ± 3.3	0.36
Ejection fraction (%)	59.7 ± 9.0	60.0 ± 9.0	59.1 ± 9.2	0.59
***Cardiovascular risk factors***				
Hypertension	100 (72.5%)	68 (73.1%)	32 (71.1%)	0.81
Diabetes mellitus	50 (36.2%)	31 (33.3%)	19 (42.2%)	0.31
Hyperlipidemia	11 (8.0%)	8 (8.6%)	3 (6.7%)	0.70
Current smoker	36 (26.1%)	25 (26.9%)	11 (24.4%)	0.75
Previous percutaneous coronary intervention	56 (40.6%)	35 (37.6%)	21 (46.7%)	0.30
Multivessel disease	84 (60.9%)	57 (61.3%)	27 (60.0%)	0.88
SYNTAX score	19.3 ± 8.9	19.7 ± 8.8	19.1 ± 9.2	0.71
***Hemodynamic parameters***				
Systolic blood pressure (mmHg)	130.1 ± 19.2	129.1 ± 19.2	132.3 ± 17.9	0.35
Diastolic blood pressure (mmHg)	78.6 ± 12.6	77.8 ± 12.4	79.3 ± 12.2	0.50
***Discharge medication***				
Aspirin	138 (100.0%)	93 (100.0%)	45 (100.0%)	NA
P2Y_12_ inhibitor	138 (100.0%)	93 (100.0%)	45 (100.0%)	NA
Beta-blocker	88 (63.8%)	59 (63.4%)	29 (64.4%)	0.91
RAAS blockade	75 (54.3%)	53 (57.0%)	22 (48.9%)	0.37
Statin	138 (100.0%)	93 (100.0%)	45 (100.0%)	NA
**Lesion characteristics**				
***Target vessel***				
LAD	171 (55.3%)	126 (56.0%)	45 (53.6%)	0.79
LCX	50 (16.2%)	32 (14.2%)	18 (21.4%)	0.29
RCA	88 (28.5%)	67 (29.8%)	21 (25.0%)	0.56
***Procedural characteristics***				
Pre-PCI diameter stenosis	76.8 ± 9.7	77.2 ± 8.9	76.4 ± 10.0	0.64
Pre-PCI lesion length	26.7 ± 10.9	27.9 ± 10.2	26.0 ± 12.3	0.34
Post-PCI diameter stenosis	3.1 ± 9.3	2.8 ± 7.7	3.2 ± 9.6	0.79
Total number of stents	1.3 ± 1.0	1.3 ± 1.0	1.1 ± 0.9	0.26
Mean stent diameter	3.0 ± 0.4	3.0 ± 0.4	3.1 ± 0.3	0.14
Total length of stents	40.9 ± 24.6	42.5 ± 25.7	37.1 ± 21.9	0.23
***Angiography-derived Physiologic Indices***				
Angiography-derived FFR, Post-PCI	0.91 ± 0.06	0.89 ± 0.06	0.94 ± 0.03	<0.001
Angiography-derived IMR, U	22.8 ± 10.1	17.1 ± 4.2	34.4 ± 9.0	<0.001

*Values are mean ± standard deviations or n (%)*.

*FFR, fractional flow reserve; IMR, index of microcirculatory resistance; LAD, left anterior descending artery; LCX, left circumflex artery; PCI, percutaneous coronary intervention; RAAS, renin–angiotensin–aldosterone system; RCA, right coronary artery*.

### Prognostic Implication of Angio-IMR in CAD Patients Received PCI

**Figure 5:**
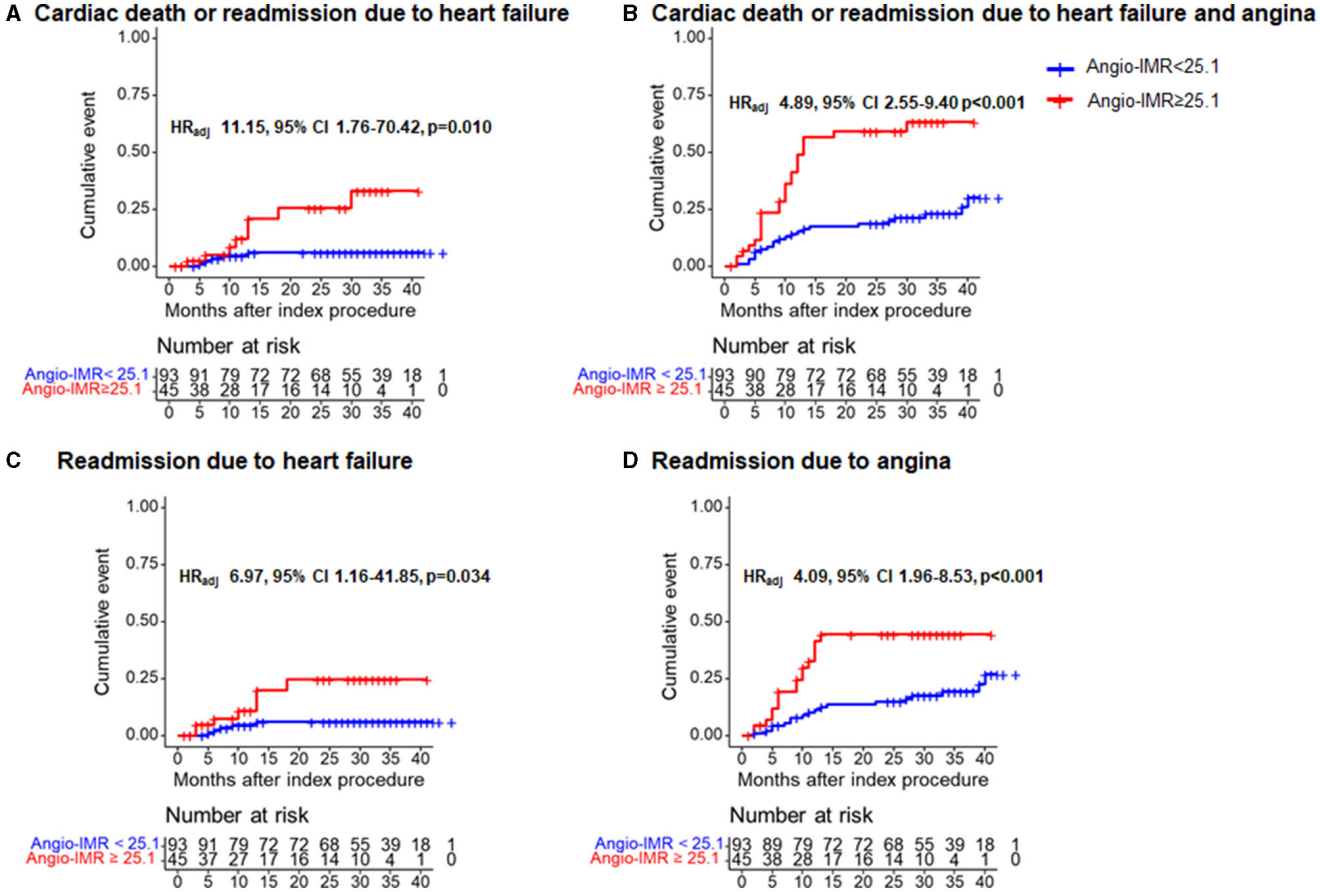
Comparison of primary and secondary outcomes at 28 months after index procedure according to angiography-derived IMR. Cumulative incidences of cardiac death or readmission due to **(A)** heart failure; **(B)** cardiac death or readmission due to heart failure and angina; **(C)** readmission due to heart failure; and **(D)** readmission due to angina at 28 months are presented according to the best cut-off value of angio-IMR. CI, confidence intervals; HR_adj_, multivariable adjusted hazard ratios; others are with Figure 1.

During a median of 28-month follow-up after index procedure, patients with angio-IMR ≥25.1 demonstrated a significantly higher incidence of cardiac death or readmission due to heart failure than those with angio-IMR <25.1 (18.6 vs. 5.4%, adjusted HR 9.66, 95% CI 2.04–45.65, *p* = 0.004) (Table 2 and Figure 5).

**Table 2:** Clinical outcomes at 28 months after index procedure according to angiography-derived IMR.

	**Angiography-derived IMR <25.1 (*N* = 93)**	**Angiography-derived IMR ≥ 25.1 (*N* = 45)**	**Univariable HR (95% CI)**	Multivariable HR (95% CI)^*^	***p*-value**
Cardiac death or readmission due to heart failure	5 (5.4%)	8 (18.6%)	5.24 (1.69–16.19)	9.66 (2.04–45.65)	0.004
Cardiac death	1 (1.1%)	2 (4.4%)	7.58 (0.67–86.23)	7.26 (0.55–95.57)	0.13
Readmission due to heart failure	6 (6.5%)	7 (15.6%)	4.28 (1.35–13.62)	5.93 (1.37–25.77)	0.02
Myocardial infarction	2 (2.2%)	2 (4.4%)	3.49 (0.48–25.65)	5.80 (0.49–68.99)	0.16
Ischemia-driven revascularization	7 (7.5%)	6 (13.3%)	2.46 (0.82–7.41)	3.20 (0.86–11.96)	0.08
Readmission due to angina	18 (19.4%)	21 (46.7%)	3.19 (1.61–6.29)	3.66 (1.68–7.97)	0.001

*The cumulative incidences of clinical outcomes are presented as event number and Kaplan–Meier estimates at 28 months after index procedure. p values were log-rank p value in survival analysis*.

* *Covariables which were included in the multivariable adjusted Cox regression model were age, sex, left ventricular ejection fraction, and post-PCI angiography-derived FFR*.

*CI, confidence intervals; HR, hazard ratios; others as in Table 1*.

The significantly higher risk of cardiac death or readmission due to heart failure in the angio-IMR ≥25.1 group was mainly due to increased risk of readmission due to heart failure than the angio-IMR<25.1 group; in addition, a higher risk of readmission due to angina was observed in patients with IMR ≥25.1, while the risk of cardiac death, MI, and ischemia-driven revascularization was similar between the two groups (Table 2). In a multivariable model, angio-IMR ≥25.1 was an independent predictor for cardiac death or readmission due to heart failure (HR 11.15, 95% CI 1.76–70.42, *p* = 0.010) (Table 3). As for the discriminant ability for cardiac death or readmission due to heart failure, angio-IMR did not increase discriminant and reclassification indices when added to the model with clinical risk factors, while for the outcomes of cardiac death or readmission due to heart failure and angina, angio-IMR increased the discriminant ability (AUC 0.71 vs. 0.53, *p* = 0.002; IDI 0.16, *p* = 0.01; category-free NRI 0.46, *p* = 0.010) (Supplementary Figure 2).

**Table 3:** Independent predictors for cardiac death or readmission due to heart failure.

**Variable**	**Univariable analysis**		**Multivariable analysis**	
	**HR (95% CI)**	***p*-value**	**HR (95% CI)**	***p*-value**
Angio-IMR ≥25.1	5.00 (1.62–15.45)	0.005	11.15 (1.76–70.42)	0.01
Age (per 10 years)	1.01 (0.95–1.08)	0.69	1.08 (0.98–1.20)	0.14
Female	2.50 (0.56–11.29)	0.23	1.93 (0.29–13.07)	0.50
Diabetes mellitus	2.11 (0.71–6.27)	0.18	2.47 (0.65–9.38)	0.19
Hyperlipidemia	2.20 (0.49–9.93)	0.31	6.78 (0.90–51.35)	0.06
Hypertension	2.17 (0.48–9.77)	0.32	0.74 (0.09–6.01)	0.78
Left ventricular ejection fraction (per 10% increase)	0.86 (0.81–0.90)	<0.001	0.84 (0.79–0.90)	<0.001
Post-PCI angio-FFR in culprit vessel ≤0.80	0.30 (0.07–1.36)	0.12	0.25 (0.02–3.11)	0.28

*Abbreviations as in Tables 1, 2*.

The best cut-off value of angio-IMR to predict the risk of cardiac death or readmission due to heart failure was 27.3 (Supplementary Figure 3). When we apply 27.3 as the cut-off to define microcirculatory dysfunction, our primary result of cardiac death or readmission due to heart failure remains unchanged (Supplementary Figure 4).

## Discussion

**Figure 6:**
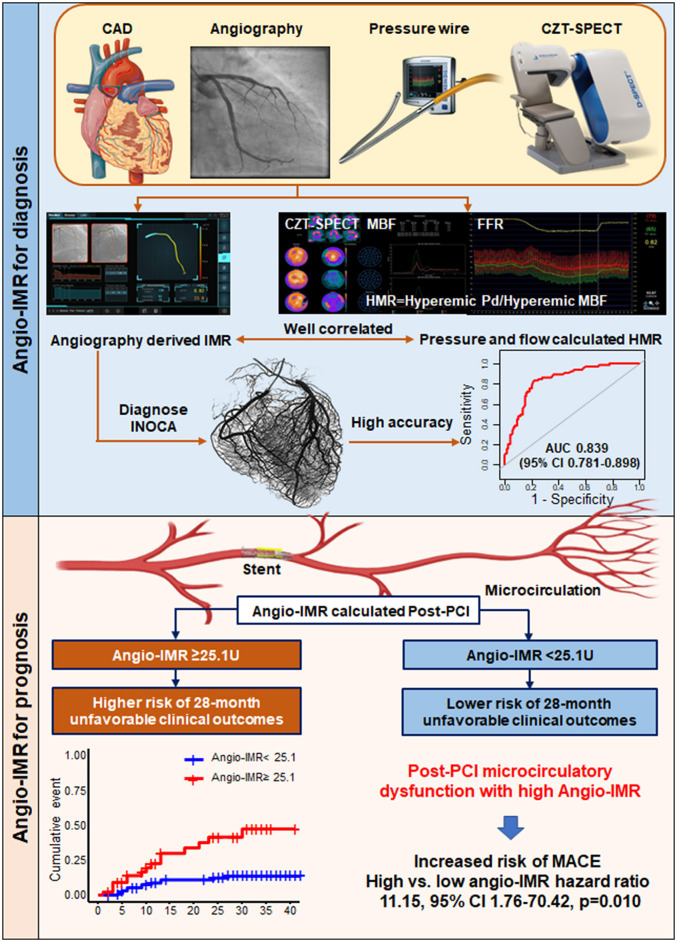
Diagnostic Value of Angiography-derived Index of Microcirculatory Resistance for Coronary Microcirculation and Its Prognostic Implication after PCI in CAD Patients. The current study evaluated diagnostic and prognostic implications of angiography-derived IMR. In diagnostic cohorts, angio-IMR showed a close correlation with HMR calculated as the ratio of hyperemic coronary pressure to myocardial blood flow, and a high diagnostic accuracy to predict patients with microcirculatory dysfunction. In prognostic cohort, patients with post-PCI impaired microcirculatory function assessed by angio-IMR ≥ 25.1U showed significantly higher risk of cardiac death or readmission due to heart failure than those with preserved microcirculatory function assessed by angio-IMR <25.1U. Angio-IMR ≥ 25.1U was independently associated with the occurrence of cardiac death or readmission due to heart failure. MACE, major adverse cardiac events; others are with Figures 1, 2.

The current study evaluated the diagnostic and prognostic implications of angio-IMR in CAD patients (Figure 6). Our main findings are as follows. First, angio-IMR showed a moderate correlation with HMR derived from coronary pressure and MBF data; second, angio-IMR showed a high diagnostic accuracy for microcirculatory dysfunction determined by normal coronary angiography and perfusion defect by CZT-SPECT; and third, patients with post-PCI angio-IMR ≥25.1 showed a significantly higher risk of cardiac death or readmission due to heart failure than those with angio-IMR <25.

### Post-PCI Microcirculatory Dysfunction in Patients With Stable CAD

Since the first PCI performed in 1977, it has been aimed to restore blood flow by relieving obstruction to the epicardial vessels. However, post-PCI angina and/or ischemia may recur or persist in a significant patient subset (16), with reported rates ranging from 15% to more than 50% (17, 18). As demonstrated in our study, 108 (78.3%) patients achieved post-PCI angio-FFR over 0.90, while there were 45 (32.6%) patients who demonstrated microcirculatory dysfunction with high angio-IMR. These findings have also been confirmed in other studies that adopted modern therapeutic strategies (19). Most importantly, symptom and/or ischemia recurrence is associated with adverse cardiovascular events (20). Accordingly, the current European Society of Cardiology guidelines on stable CAD have emphasized the importance of coronary vascular dysfunction in causing angina post-PCI (21).

### Assessment of Microvascular Disease Using Angiography-Derived IMR as an Alternative to Wire-Derived IMR

The rationale for identifying microvascular dysfunction is to provide a definitive diagnosis; then, a possible treatment may be anticipated (22). Positron emission tomography remains the reference standard for assessing myocardial blood flow (23). Unfortunately, most patients who present to the cardiac catheterization laboratory did not evaluate their microcirculation. Angiographic techniques have their limitations considering the qualitative and subjective nature. Doppler wire–derived coronary flow reserve has been applied in research studies, but its clinical role has been limited by the technical issues.

IMR is a quantitative method for specifically assessing the microvascular function of the interrogated vessel (2). The emerging data demonstrate its role in evaluating patients with chest pain and non-obstructive coronary artery disease, as well as in predicting adverse events. However, it is hampered by the need of extra care to ensure maximal hyperemia (drug type, dose, infusion route, contraindication for drug, etc.) and continuous infusion of intravenous adenosine may raise potential safety concerns. HMR is also a quantitative index for microcirculatory dysfunction; however, measuring HMR is probably more challenging than measuring IMR, with higher failure rates related to the contemporary measurement of myocardial blood flow and coronary pressure. For these reasons, there is a need for an invasive technique to rapidly, reliably, and relatively easily assess for microcirculatory dysfunction in the cardiac catheterization laboratory.

With the technical development, angiographic derivation of IMR without pressure wire, hyperemic agents, or thermodilution method is available as a potential alternative for pressure wire–derived IMR. In the internal diagnostic cohort of our study, our analysis demonstrated that angio-IMR had a significant correlation with HMR (R = 0.74, P <0.001). Furthermore, in the external diagnostic cohort, angio-IMR demonstrated a good diagnostic accuracy for microvascular disease with an AUC of 0.839 and a diagnostic accuracy of 79.8%. With no additional angiogram imaging acquisition or need for a hyperemic agent, angio-IMR may represent a promising measure as an alternative to wire-derived IMR and potentially increase the adoption of the physiological assessment of microvascular diseases in the cardiac catheterization lab.

### Prognostic Implication of Microcirculatory Dysfunction in Patients Received PCI

Microvascular disease has been confirmed to be associated with a higher risk of cardiovascular events in patients without obstructive epicardial stenosis (24, 25). In our study, the prognostic implication of angio-IMR in patients after PCI was evaluated. Increased angio-IMR was significantly associated with the higher risk of cardiac death or readmission due to heart failure and incidence of angina. In multivariable analysis, increased angio-IMR ≥25.1 remained as an independent predictor for cardiac death or readmission due to heart failure. These results are in line with the previous studies. Studies by Fearon et al. and Carrick et al. have shown that high IMR after primary percutaneous coronary intervention predicts adverse clinical outcomes in patients with myocardial infarction (26, 27). A recent study indicated that IMR measured immediately after PCI predicts adverse events in patients with stable CAD (28).

Our study provided a simple and convenient quantitative index to assess patients' microcirculatory status at the time of PCI. Further research is needed to assess whether angio-IMR-guided strategies might improve prognosis in patients with microcirculation dysfunction compared with standard care.

## Limitations

There are some limitations that should be considered. First, because of the comprehensive study protocol, the number of patients included in our study was limited; our findings need to be verified in other cohorts with a larger sample size. Second, as a retrospective study, our included patients did not receive IMR assessment, which is a “gold standard” for microvascular dysfunction; third, though we observed a moderate correlation between angio-IMR and HMR, no definite normal range for HMR was reported yet. As a complementary, we investigated the diagnostic performance of angio-IMR in INOCA patients and angio-IMR showed a high accuracy for predicting microcirculatory dysfunction. Fourth, ROC analysis determined the best cut-off value for angio-IMR as 25.1, which is very close to that for pressure wire–measured IMR. Thus, we used this cut-off value to define post-PCI microcirculatory dysfunction. However, the underlying mechanism between INOCA and post-PCI patients may be different. We further used the maximally selected log-rank statistics method to derive the best cut-off value of angio-IMR to predict clinical outcomes as 27.3; when we used this value as a cut-off, our primary results remained unchanged.

## Conclusions

In conclusion, this study demonstrated that angiography-derived IMR had a moderate correlation with HMR derived by CZT-SPECT and pressure-wire measurement and a good diagnostic accuracy to predict microcirculatory dysfunction. An elevated angio-IMR measured at the time of PCI predicts a higher risk of cardiac death or heart failure admission at 28 months.

## Data Availability Statement

The raw data supporting the conclusions of this article will be made available by the authors, without undue reservation.

## Ethics Statement

The studies involving human participants were reviewed and approved by Zhongshan Hospital Fudan University and Shanghai Tenth People's Hospital. The patients/participants provided their written informed consent to participate in this study.

## Author Contributions

ND, WC, and LL drafted and revised the manuscript. WZ, GY, BX, YX, CL, KY, and DH collected the data and follow up the patients. SD and HY analyzed the data and did the statistical analysis. JG designed the study. All authors contributed to the article and approved the submitted version.

## Funding

This study was partly supported by National Key R&D Program of China 2016YFC1301203 and 2016YFC1301200. ND was funded by project 81600279 from the National Natural Science Foundation of China and project 2019M650075 from China Postdoctoral Science Foundation.

## Conflict of Interest

The authors declare that the research was conducted in the absence of any commercial or financial relationships that could be construed as a potential conflict of interest.

## Publisher's Note

All claims expressed in this article are solely those of the authors and do not necessarily represent those of their affiliated organizations, or those of the publisher, the editors and the reviewers. Any product that may be evaluated in this article, or claim that may be made by its manufacturer, is not guaranteed or endorsed by the publisher.

## Abbreviations

Angio-IMR: angiography-derived index of microcirculatory resistance
Angio-FFR: angiography-derived fractional flow reserve
CAD: coronary artery disease
CZT-SPECT: cadmium–zinc–telluride single-photon emission computed tomography
FFR: fractional flow reserve
HMR: hyperemic microvascular resistance
MBF: myocardial blood flow
IMR: index of microcirculatory resistance
INOCA: ischemia and no obstructive coronary artery disease
PCI: percutaneous coronary intervention.
