# Supplementary material for: Diagnostic Value of Angiography-Derived IMR for Coronary Microcirculation and Its Prognostic Implication After PCI
Source: Front Cardiovasc Med. 2021 Oct 15;8:735743. doi: 10.3389/fcvm.2021.735743 (PMC8553988; doi:10.3389/fcvm.2021.735743)

**Diagnostic Value of Angiography-derived Index of Microcirculatory Diagnostic Value of Angiography-derived IMR for Coronary Microcirculation and Its Prognostic Implication after PCI**

Neng Dai, MD^a,b^**^✝^**, Wenliang Che, MD, PhD^c^**^✝^**, Lu Liu, MD^c^**^✝^**, Wen Zhang, MD^c^, Guoqing Yin, MD^c^, Bin Xu, MD^c^, Yawei Xu, MD, PhD^c^, Shaofeng Duan, PhD^d^, Haojun Yu, BS^e^, Chenguang Li, MD^a,b^, Kang Yao, MD^a,b^, Dong Huang, MD, PhD^a,b^, Junbo Ge, MD^a,b^

^a^Department of Cardiology, Zhongshan Hospital, Fudan University, Shanghai Institute of Cardiovascular Diseases, Shanghai, China;

^b^National Clinical Research Center for Interventional Medicine, Shanghai, China;

^c^Department of Cardiology, Shanghai Tenth people’s Hospital, Tongji University, Shanghai Institute of Cardiovascular Diseases, Shanghai, China;

^d^GE Healthcare China, Shanghai, China;

^e^Department of Nuclear Medicine, Zhongshan Hospital, Fudan University, Shanghai, China

**^✝^Contributed equally to this work.**

**Corresponding author:**

Junbo Ge, MD

**Table of Contents**

- **Supplemental Tables**
- **Supplemental Figures and Figure Legends**

**Supplemental Tables**

**Supplemental Table 1.** **Patient and Lesion Characteristics of Internal Diagnostic Cohort**

| **Patient characteristics** |  | **Lesion characteristics** |  |
| --- | --- | --- | --- |
| ***Demographics*** | ***N=53*** | ***Quantitative coronary angiography*** | ***N=53*** |
| Age (years) | 63.5±9.4 | Reference vessel diameter, mm | 2.6±0.4 |
| Male | 48(90.6%) | Minimal lumen diameter, mm | 1.5±0.4 |
| Body mass index (kg/m^2^) | 24.6±2.1 | Diameter stenosis, % | 44.4±12.0 |
| Ejection fraction (%) | 60.2±5.7 | Lesion length, mm | 11.9±8.9 |
| ***Cardiovascular risk factors*** |  | Vessel SYNTAX score | 12.5±8.0 |
| Diabetes mellitus | 17(32.1%) | ***Physiological Index*** |  |
| Hypertension | 37(69.8%) | Angiography derived FFR | 0.84±0.06 |
| Hyperlipidemia | 45(84.9%) | Angiography derived IMR | 24.7±3.2 |
| Smoking | 29(54.7%) | Pressure wire-derived FFR | 0.81±0.06 |
| Family history of coronary artery disease | 8(15.1%) | Hyperemic Myocardial blood flow | 1.94±0.43 |
| Previous myocardial infarction | 4(7.5%) | Resting Myocardial blood flow | 0.90±0.19 |
| ***Hemodynamic parameters*** |  | Myocardial flow reserve | 2.21±0.61 |
| Systolic blood pressure (mmHg) | 124.0±16.1 | Hyperemic microcirculatory resistance | 40.29±10.94 |
| Diastolic blood pressure (mmHg) | 79.3 ±10.3 | Resting microcirculatory resistance | 106.74±23.45 |

Values are mean ± standard deviations or n (%).

FFR = fractional flow reserve; IMR = index of microcirculatory resistance; SYNTAX = Synergy Between Percutaneous Coronary Intervention with Taxus and Cardiac Surgery;

**Supplemental Table2. Patient Characteristics of External Diagnostic Cohort**

| **INOCA Patients (N=35)** |  | **Normal controls (N=45)** |  |
| --- | --- | --- | --- |
| ***Demographics*** |  | ***Demographics*** |  |
| Age (years) | 60.6±8.6 | Age (years) | 60.7±9.0 |
| Male | 8 (22.9%) | Male | 22 (48.9%) |
| Body mass index (kg/m^2^) | 25.8±3.0 | Body mass index (kg/m^2^) | 24.6 ±3.1 |
| Ejection fraction (%) | 63.5±3.9 | Ejection fraction (%) | 63.6±2.9 |
| ***Cardiovascular risk factors*** |  | ***Cardiovascular risk factors*** |  |
| Diabetes mellitus | 4 (11.4%) | Diabetes mellitus | 7(15.6%) |
| Hypertension | 19(54.3%) | Hypertension | 21(46.7%) |
| Hyperlipidemia | 1(2.9%) | Hyperlipidemia | 6(13.3%) |
| Smoking | 3(8.6%) | Smoking | 8(17.7%) |
| ***Physiological Index*** |  | ***Physiological Index*** |  |
| SSS (Median ± IQR) | 4±2 | SSS (Median ± IQR) | 0±0 |
| SRS (Median ± IQR) | 0±0 | SRS (Median ± IQR) | 0±0 |
| SDS (Median ± IQR) | 3±2 | SDS (Median ± IQR) | 0±0 |
| Angiography-derived FFR | 0.94±0.03 | Angiography-derived FFR | 0.93±0.03 |
| Angiography-derived IMR, U | 35.83±13.35 | Angiography-derived IMR, U | 23.7±9.0 |

Values are mean ± SD or n (%), or Median± IQR.

FFR = fractional flow reserve; IMR = index of microcirculatory resistance; INOCA = ischemia and no obstructive coronary artery disease; IQR = interquartile range; SDS = summed difference score; SRS = summed rest score; SSS = summed stress score

**Supplemental Figures and Figure Legends**

**Supplemental Figure 1. Correlation and agreement between 2 repeated measurements of Angiography-Derived FFR and IMR**

The correlation and agreement between 2 repeated measurements of (A) angio-FFR and (B) angio-IMR.

Abbreviations: Angio-FFR1, first measurement of angiography derived fractional flow reserve; Angio-FFR2, second measurement of angiography derived fractional flow reserve; Angio-IMR1, first measurement of angiography derived index of microcirculatory resistance; Angio-IMR2, second measurement of angiography derived index of microcirculatory resistance; SD, standard deviation.


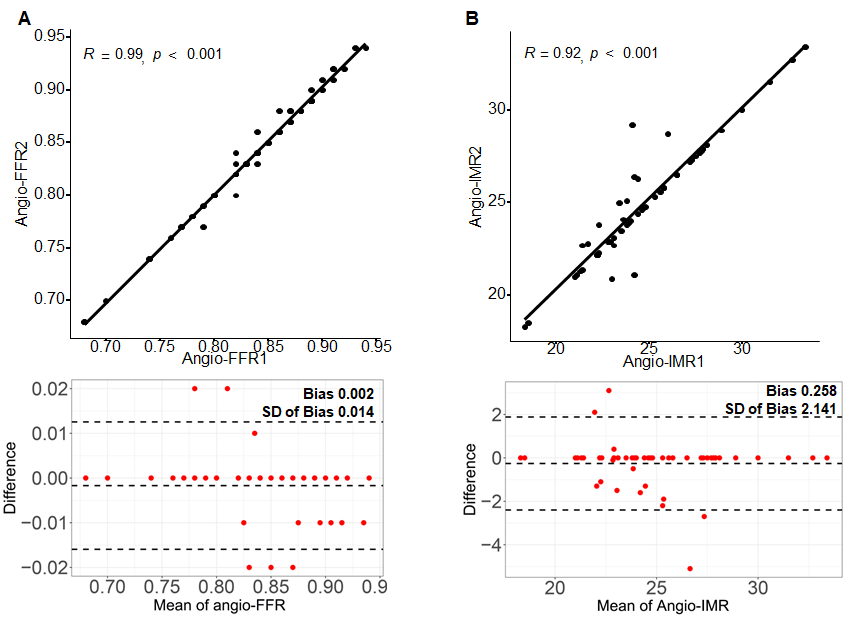


**Supplemental Figure 2. Incremental Prognostic Value of Angiography-Derived IMR over Clinical and Angiographic Factors**

Discriminant functions to predict 28-month (A) cardiac death or readmission due to heart failure and (B) cardiac death or readmission due to heart failure and angina are presented. The reference model included clinical risk factors only, including age, sex, LVEF, and post-PCI angio-FFR. The model with angio-IMR significantly increased discriminant and reclassification abilities for predicting clinical outcomes of cardiac death or readmission due to heart failure and angina than the reference model.

Abbreviations: AUC, area under curve; IDI, integrated discrimination improvement; NRI, net reclassification index.


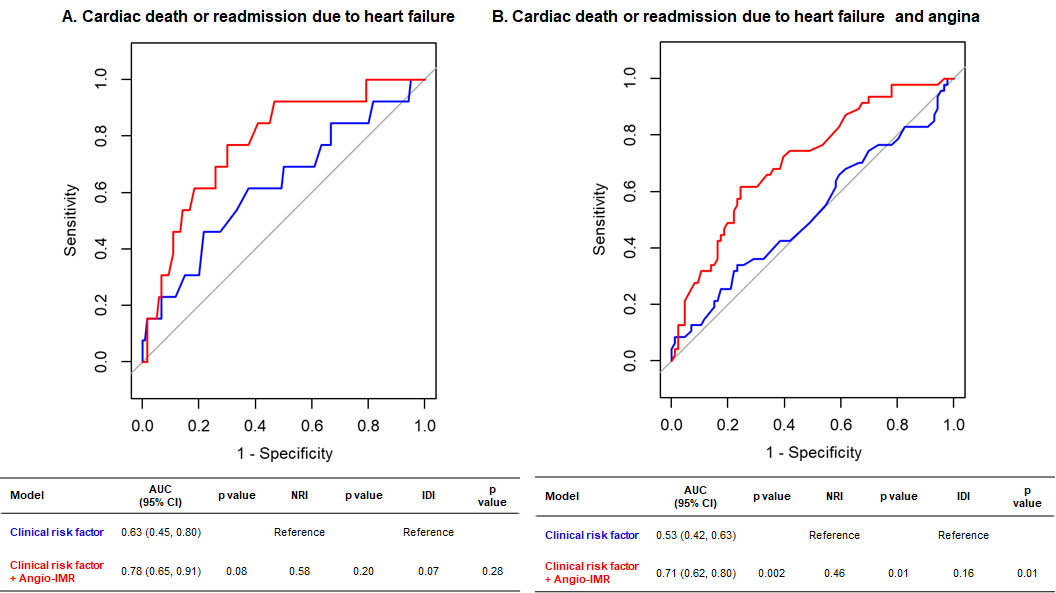


**Supplemental Figure 3. Best Cut-Off Value of Angio-IMR for Cardiac Death or Readmission due to Heart Failure**

Best cut-off value of angio-IMR to predict the risk of cardiac death or readmission due to heart failure which was evaluated by the maximally selected log-rank statistics method. The best cut-off value of angio-IMR was >27.3 U.

Abbreviations: Angio-IMR, angiography derived index of microcirculatory resistance


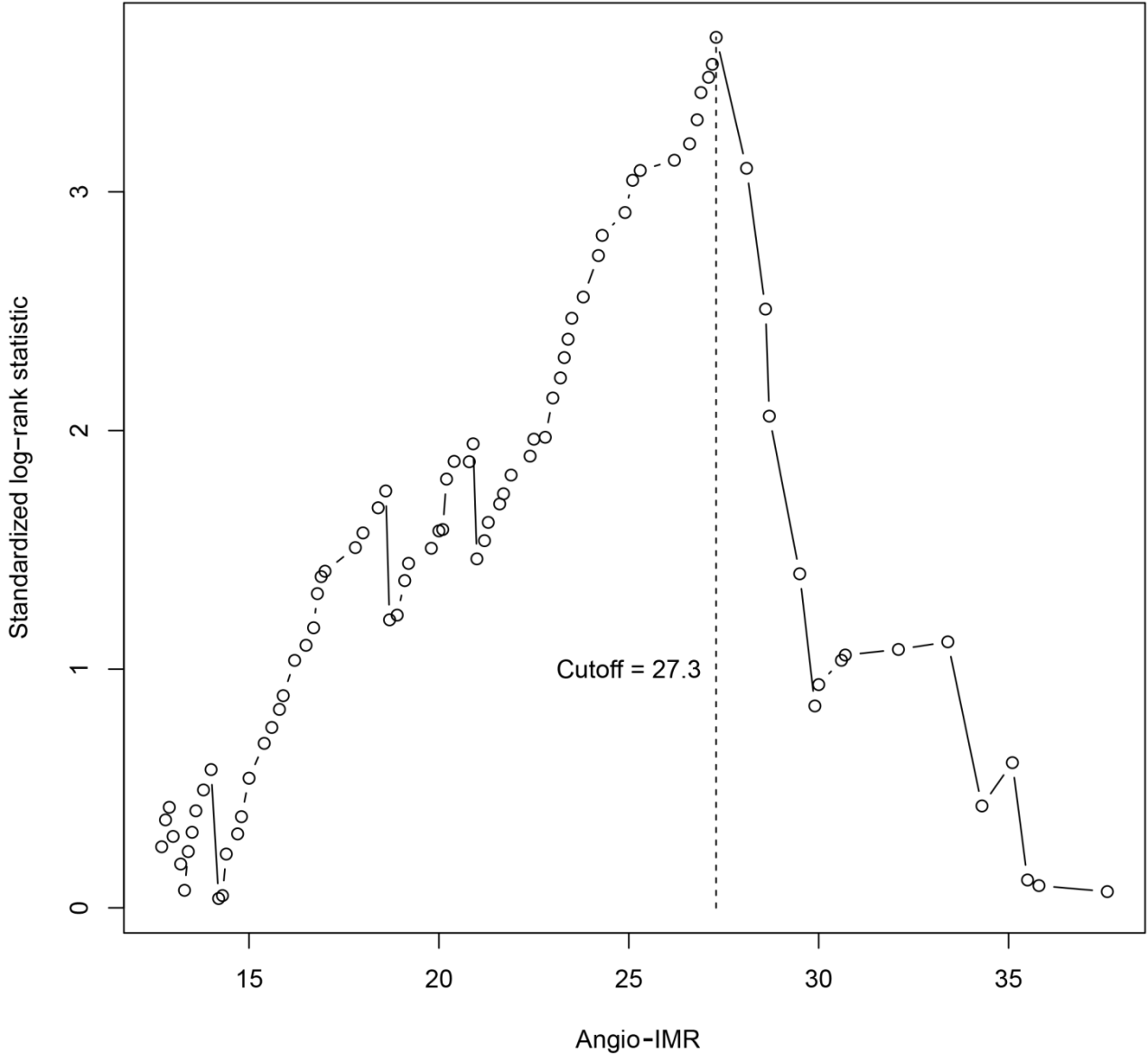


**Supplemental Figure 4. Sensitivity Analysis for Primary and Secondary Outcomes at 28 Months After Index Procedure According to Angiography-Derived IMR with Cut-off Value of 27.3**

Cumulative incidences of (A) cardiac death or readmission due to heart failure; (B) cardiac death or readmission due to heart failure and angina; (C) readmission due to heart failure; and (D) readmission due to angina at 28 months are presented according to the best cut-off value of angio-IMR.

Abbreviations: CI, confidence intervals; HR_adj_, multivariable adjusted hazard ratios;


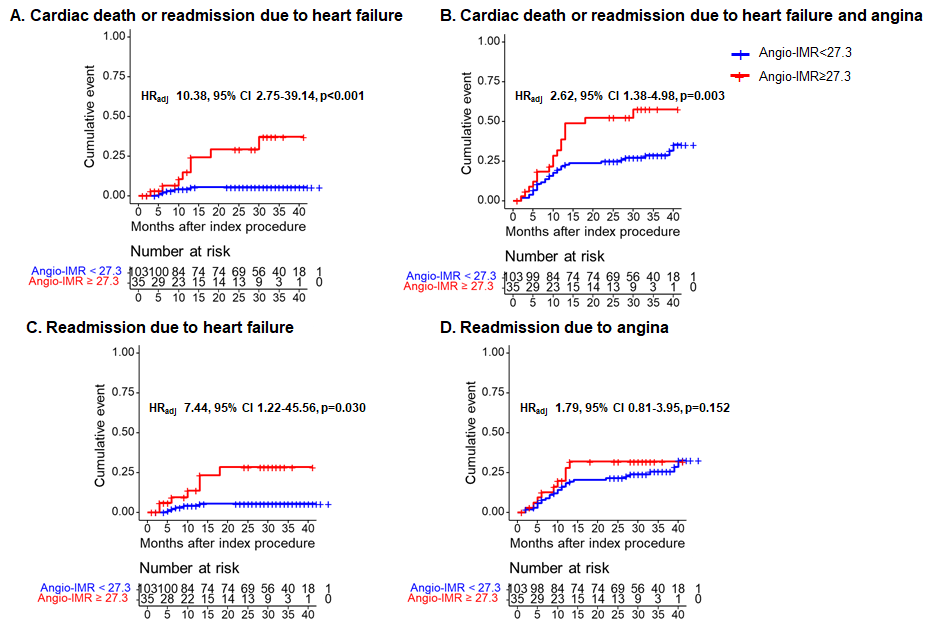

Supplement: Supplementary file 1 [file Data_Sheet_1.docx]
